# Finger Nails

**Published:** 1878-06

**Authors:** 


### SUGGESTIVE.

### FINGER NAILS.

The finger nails grow their whole length in a few months —faster in summer than in winter. They should be trimmed once a week slowly and composedly, with a pair of sharp blunt-end scissors, closer at the ends than at the sides, thus preventing the skin from rising, causing as to the toes, that most distressing ailment called in-growing toe nail. Accumulations under the ends of the nails should not be removed by any metallic substance, but by a piece of soft wood, or which is still better, by a thorough washing of the hands. We have seen "beauty and the beast" united, in a handsome face, and a suggestive rim of black at the ends of the fingers.

